# The Impact of Nanoparticles on Innate Immune Activation by Live Bacteria

**DOI:** 10.3390/ijms21249695

**Published:** 2020-12-18

**Authors:** Benjamin J. Swartzwelter, Alexandra C. Fux, Litty Johnson, Elmer Swart, Sabine Hofer, Norbert Hofstätter, Mark Geppert, Paola Italiani, Diana Boraschi, Albert Duschl, Martin Himly

**Affiliations:** 1 Department of Biosciences, Paris Lodron University of Salzburg (PLUS), 5020 Salzburg, Austria; swartzwe@colorado.edu (B.J.S.); alexandra.fux@sbg.ac.at (A.C.F.); litty.johnson@sbg.ac.at (L.J.); sabine.hofer@sbg.ac.at (S.H.); norbert.hofstaetter@sbg.ac.at (N.H.); mark.geppert@sbg.ac.at (M.G.); albert.duschl@sbg.ac.at (A.D.); 2 Institute of Biochemistry and Cell Biology, National Research Council, 80131 Napoli, Italy; paola.italiani@ibbc.cnr.it (P.I.); diana.boraschi@ibbc.cnr.it (D.B.); 3 UK Centre for Ecology and Hydrology (UKCEH), Wallingford OX10 8BB, UK; elmswa@ceh.ac.uk; 4 Stazione Zoologica Anton Dohrn, 80122 Napoli, Italy

**Keywords:** engineered nanoparticles, innate immunity, inflammation, innate immune memory, toll-like receptors, pathogen-associated molecular patterns, lipopolysaccharide, biocorona, microbiota, nanovaccines, adjuvants

## Abstract

The innate immune system evolved to detect and react against potential dangers such as bacteria, viruses, and environmental particles. The advent of modern technology has exposed innate immune cells, such as monocytes, macrophages, and dendritic cells, to a relatively novel type of particulate matter, i.e., engineered nanoparticles. Nanoparticles are not inherently pathogenic, and yet cases have been described in which specific nanoparticle types can either induce innate/inflammatory responses or modulate the activity of activated innate cells. Many of these studies rely upon activation by agonists of toll-like receptors, such as lipopolysaccharide or peptidoglycan, instead of the more realistic stimulation by whole live organisms. In this review we examine and discuss the effects of nanoparticles on innate immune cells activated by live bacteria. We focus in particular on how nanoparticles may interfere with bacterial processes in the context of innate activation, and confine our scope to the effects due to particles themselves, rather than to molecules adsorbed on the particle surface. Finally, we examine the long-lasting consequences of coexposure to nanoparticles and bacteria, in terms of potential microbiome alterations and innate immune memory, and address nanoparticle-based vaccine strategies against bacterial infection.

## 1. Introduction

### 1.1. The Human Innate Immune Response

The innate immune response begins with cellular or humoral recognition of danger, which alerts cells of the presence of potentially pathogenic foreign or endogenous materials. Upon recognition of danger, innate immune cells such as monocytes and macrophages rapidly initiate a response that includes phagocytosis and leads to inflammation, which are aimed at removal and destruction of the threatening object [1,2]. Activation of innate cells is generally initiated by pathogen binding to pattern recognition receptors (PRRs) expressed on the plasma membrane and on intracellular membranes. Ligand binding of PRRs initiates signalling pathways that lead to production and release of inflammatory factors, such as cytokines and chemokines, and upregulation of costimulatory surface molecules [3]. The classic example of a PRR-activating ligand is lipopolysaccharide (LPS), which is present on Gram-negative bacteria and activates the PRR toll-like receptor 4 (TLR4) [4,5]. Different pathogen-associated molecular patterns (PAMPs) bind other PRRs and lead to a similar inflammatory cell activation. Due to the fact that they potently stimulate innate immune responses, PRR ligands such as LPS are routinely used for in vitro activation and assessment of innate/inflammatory responses, particularly to examine the impact of drug candidates and biological materials [6]. In in vitro testing of novel biomedical products, the reaction of innate immune cells to LPS or other PAMPs is generally considered as a proxy for the general reaction to pathogenic stimulation, in order to obtain insight into whether a material may have a direct impact on or interfere with a normal innate immune response. Several important limitations of this experimental/screening approach should be considered. Innate immune cells typically come in contact with bacteria after they have crossed protective barriers such as the intestinal or respiratory epithelium, and thus in vivo the interaction mostly occurs with whole live bacteria. While PRR activation is a central component to the resulting immune response, innate cells also respond to other important physical and active aspects of bacterial invasion such as the shape and size, and the spatial organization of PAMPs on the bacterial surface [7,8]. Additionally, bacterial motility and proliferation can dramatically impact defensive cell activation [9]. In vitro models that attempt to simulate the in vivo response should therefore consider the use of whole and live bacteria, to more completely mimic the processes inherent to an immune response to infection. Based on such considerations, a re-evaluation of the immunological effects of engineered nanoparticles (NPs) may be necessary, as the majority of nanoparticle related studies do not address the impact of nanoparticles in the context of challenge by live bacteria.

### 1.2. Current State of Nanotechnology from a Biological Perspective

Nanotechnology is one of the key technologies of the 21st century with many applications, including in biomedical sciences. NPs (i.e., particles with dimensions of 1–100 nm) can be composed of different materials and can appear in different sizes, shapes, and with different surface functionalization. Silicon dioxide (SiO_2_), titanium dioxide (TiO_2_), and zinc oxide (ZnO) NPs are the most produced types of NPs worldwide [10]. Applications can be found in electronics (SiO_2_), manufacturing and construction (e.g., car tires, concrete, sports equipment; SiO_2_, TiO_2_, and also aluminum oxide, Al_2_O_3_, and carbon nanotubes or graphene), food additives (SiO_2_ and TiO_2_), food contact materials or textiles (silver, Ag), paintings (TiO_2_), and sunscreens (TiO_2_ and ZnO) among others [11,12,13]. Despite their broad applications, there is evidence that certain NPs can induce cell stress and/or toxicity or cause unwanted immune reactions [14,15]. Thus, a manifold of “safe-by-design” strategies have been developed during the past decades in the attempt to design safer nanomaterials [16]. From a biomedical standpoint, many NP formulations can be used for therapeutic benefit. Iron oxide NPs can function as contrast agents in magnetic resonance imaging, for tumour therapy by magnetic hyperthermia, for iron replacement therapy, and are in development for use in drug delivery [17,18,19]. Gold (Au) NPs offer a number of potential medical applications in various areas, including bioimaging, sensing, diagnosis, and functionalization for therapeutic purposes [20]. Biomedically useful organic and biodegradable NPs such as poly-lactic-co-glycolic acid (PLGA) NPs have been approved by the US Food and Drug Administration and the European Medicines Agency as drug delivery systems [21,22,23]. Nanomedical applications are also under development for passive or active drug targeting in the field of cancer treatment [24], and have great potential in vaccination [25] and immunotherapeutic approaches [26,27].

The synthesis methods for generating NPs include gas-phase and wet-chemical methods, and procedures involving organic solvents that extend even to the production of biocompatible and biodegradable nanomaterials [28,29,30,31]. Elimination of organo-chemical residues is particularly important in NPs meant for biological application. Wet-chemical methods may be especially prone to contamination of the resulting NPs with bacterial components such as endotoxin (i.e., LPS) that, even after sterilization, can induce an inflammatory response that may be mistakenly attributed to particles [32]. Nanomaterials may interact with humans at multiple levels throughout their life cycle, with exposure scenarios ranging from food additives and consumer products to the workplace and the medical field. This implies that the interaction may occur at the level of different barrier tissues and that the NP exposure doses may significantly vary. Besides human exposure, we should be aware that manufacturing, use, and disposal of such nanomaterials may be also detrimental to the environment where they may exert undesirable effects on the entire biosphere, including microorganisms [33,34]. This may again lead to indirect effects on human health by impacting biodiversity in the environment, and thus the food chain, and the microbiota in symbiosis with humans. In each case, from synthesis to environmental effects or direct particle exposure, the potential exists for nanoparticles to impact human health.

**Figure 1:**
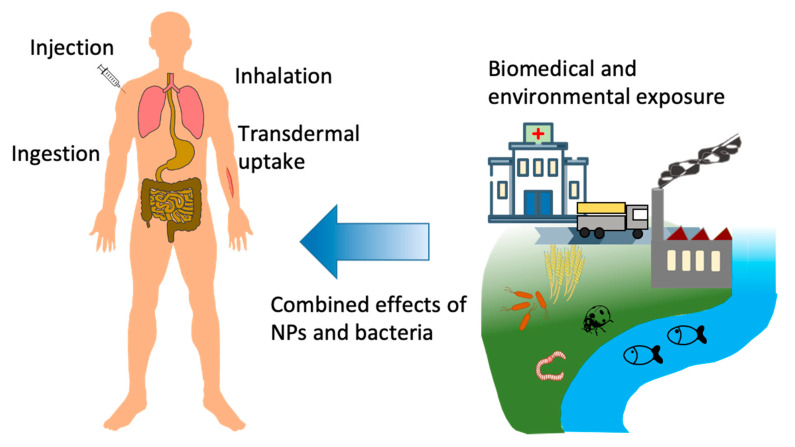
Overview of potential coexposure scenarios/interactions of nanoparticles (NPs) with ubiquitous bacteria/bacterial components, and main routes of uptake into the human body.

The direct effects of different nanomaterials on the innate immune system have been reviewed earlier [35,36,37]. NPs can enter the human body by different routes such as inhalation, ingestion, injection, or skin contact (depicted in Figure 1). Some inhaled or ingested NPs have been shown to penetrate the relevant biological barriers, the alveolar epithelium, or the intestinal epithelium [38]. While dermal penetration can be considered as a minor entry route of NPs, some studies provided evidence that NPs can to a certain extent penetrate the protective layers of the skin, in particular if the skin presents anomalies [39,40]. Once inside the body, NPs can interact with different components of the innate immune system, such as neutrophils, monocytes, macrophages, dendritic cells, natural killer cells, etc. [36,41]. Furthermore, NPs may also impact adaptive immune responses for example by modulating the function of dendritic cells in antigen presentation [35]. The question of whether or not a nanomaterial can be considered as immunologically safe is heavily discussed in the current literature and depends on numerous factors, including the condition of the target host [42,43]. For instance, elderly people or people with chronic diseases are more likely to develop detrimental reactions to NPs that pose no problem to healthy people in identical exposures [44]. Other factors depend on the nanomaterial itself (size, shape, and composition), its potential contamination (especially with LPS), its concentration upon exposure and the sensitivity of the target host [45]. While the capacity of NPs to directly impact the immune system has been extensively studied, far less literature exists on the impact of NPs upon coexposure with infectious pathogens. Within this review we focus on the capacity of NPs to affect the innate immune activation triggered by live bacteria, thereby modulating the course of a normal defensive innate/inflammatory reaction. We have chosen to focus on NPs that are not functionalized with specific molecules, in order to examine the direct impact of the bionano interaction on the antibacterial immune response. 

## 2. The Effects of NPs on Innate Immune Stimulation

Many in vitro models that investigate the interaction of novel NP formulations with innate immune activation use synthetic or purified ligands that activate monocytes, macrophages, or other PRR-expressing cells. PRR-based cell activation leads to production of molecules involved in the defensive inflammatory reaction, including costimulatory surface molecules and soluble factors such as cytokines and chemokines. Several scenarios exist in which unfunctionalized NPs may interfere with and alter both the initiation and progression of inflammatory responses induced by microbial or other stimuli. Of most concern from a pathological standpoint is the possibility that NPs could exacerbate or prolong PRR-driven inflammatory reactions, leading to uncontrolled tissue-damaging inflammation. For instance, NPs may synergize with and amplify the effects of the bacterial stimulation. An exemplary case is the production of interleukin-1β (IL-1β). LPS binding to TLR4 upregulates, through the transcription factor NF*κ*B, the gene encoding the potent inflammatory cytokine IL-1β [46]. IL-1β is produced as an inactive pro-protein that needs enzymatic cleavage before being exported extracellularly as biologically active IL-1β. This usually requires a second signal involving activation of the NOD-, LRR-, and pyrin domain-containing protein 3 (NLRP3) inflammasome, which induces the enzyme caspase-1 to cleave pro-IL-1β [47,48]. Active IL-1β then leaves the cell and binds to IL-1 receptors (which are present on most cells in the body, erythrocytes being an exception) and triggers defensive activation responses [49]. If uncontrolled in duration and intensity, IL-1β-induced inflammation can lead to pathological symptoms such as vasodilation, leukocyte influx, swelling, fever, and eventually inflammation-driven tissue damage may occur [50]. Silica NPs, multiwalled carbon nanotubes and many other NP types have been shown to be potent inducers of NLRP3 inflammasome activation, an event that could contribute to the establishment of uncontrolled pathological inflammation [51,52]. Conversely, NLRP3 activation by NPs could be exploited in a beneficial manner in the case of adjuvant function, where activation of the innate immune system is desirable for induction of long-lasting adaptive immunity [4,26]. 

Although some NPs may enhance inflammation by acting on NLRP3 activation, in several cases, it was observed that an inflammatory response to inflammatory stimuli could be downregulated by the presence of NPs. This is most commonly reported in terms of reduced cytokine production in response to LPS stimulation. Nanoplatinum was observed to suppress the production of cytokines and reactive oxygen species (ROS) in LPS-stimulated mouse macrophage-like leukemia cells [53]. Grosse et al. demonstrated an inhibition of the response to LPS in terms of production of tumor necrosis factor-alpha (TNFα), IL-1β, and IL-6 when human primary monocytes were exposed to iron oxide NPs, an effect more pronounced at higher particle concentrations but smaller particle sizes [54]. Possible explanations include a NP “cleaning” effect, in which the stimulant (as in the case of IL-1 β) is adsorbed onto the particle surface limiting its capacity to bind to its receptor [55]. Other evidence demonstrated that mouse bone marrow macrophages preexposed to superparamagnetic iron oxide NPs (SPIONs) exhibit a more inflammatory gene activation profile in response to LPS, raising another potential pathway for altering LPS-induced reactivity [56]. However, as experiments with similarly sized metallic or silica NPs failed to interfere with LPS-induced inflammation [32,56], it is likely that the NP effects may depend on the particle type and concentration, the exposure characteristics (before LPS, concurrent with LPS, etc.), the cell type (transformed vs. primary, human vs. murine) and the assay conditions. 

We noted that in many cases, exposing innate immune cells to NPs has no effect on cellular reactivity to inflammatory stimuli, in spite of the fact that cells recognize and uptake the particles to which they are exposed [6,57,58]. This phenomenon is likely widely underreported, as it is inherently a “no-result” outcome, and these data have a tendency to be omitted from the literature. We also noted that much of the literature concerning NPs and immune activation regards the mechanistic aspects of the interaction, and uses particle and stimulant doses that are poorly related to realistic human exposure scenarios [45].

Some examples for the impact of unfunctionalized NPs on innate immune activation can be found in Table 1.

## 3. The Effect of NPs on Innate Immune Activation by Live Bacteria

Activation of innate immune responses by bacteria typically occurs, as already mentioned, principally through recognition of bacterial surface patterns by PRRs, including TLRs. In addition, microorganisms that enter the cytosol can be recognized by a class of cytosolic PRRs, the NOD-like receptors (NLRs). While these are the most likely and potent mechanisms of innate immune activation, in the case of bacterial stimulation several additional variables must be considered beyond the ligand–receptor based effect. A primary task of the innate immune response is to restrict bacterial growth and spread. Malfunctioning or suppressed immune responses, for instance in the case of diabetic chronic wounds, can result in uncontrolled bacterial proliferation leading to chronic and tissue-damaging infection [67].

Upon recognition of potentially dangerous agents such as bacteria, macrophages attempt to engulf the threat for destruction and elimination by activating the energy-expensive process of phagocytosis [2]. The efficiency of cell-mediated host defense also depends upon effective and rapid capture of motile bacteria and on the motility of phagocytes themselves [68,69]. Some bacteria such as *Staphylococcus aureus* have evolved an active immune avoidance mechanism by secreting biofilms, which strongly decrease immune detection or even drive the activation of immunosuppressive cells [70,71]. Further, some bacterial species are capable of intracellular survival, residing within phagosomes and preventing their fusion with lysosomes to escape phagolysosome-based destruction [72]. These processes are dynamic, adding increased levels of physical and chemical complexity that are not mimicked by PPR activation with soluble ligands such as LPS. This underlines the significant difference, in terms of molecular and functional mechanisms engaged in the response, between ligand-induced PPR stimulation (mimicking a late response, once bacteria have been destroyed and only single molecules are still present, with only PRR stimulation still occurring), uptake of dead bacteria (mimicking an intermediate phase of the response, once bacteria have been killed extracellularly but not yet destroyed; PRR stimulation and phagocytosis still occurring), and interaction with live bacteria (mimicking the first phases of the responses, with PRR stimulation, phagocytosis, proliferation restriction, and bacterial killing occurring). Thus, in vitro assays exclusively based on PPR stimulation would only reproduce the late phase of an innate immune response to bacteria and may not be predictive of the possible interference of NPs in particular with the early innate reaction to bacteria. This becomes particularly important in scenarios where coexposure to NPs and bacteria may take place. Certainly, in consumer or occupational exposure to NPs in the respiratory or digestive tract an interaction with the microbiota colonizing the respiratory and digestive mucosae is expected [73,74]. NPs may also be applied topically for a variety of purposes, where interactions with epidermal bacteria would be unavoidable [75]. Increased use of NPs in modern society makes it likely that coexposure to bacteria and NPs will become more common.

**Figure 2:**
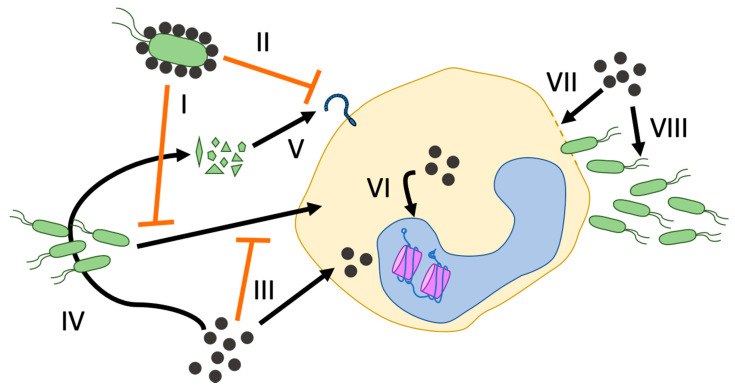
A schematic representation of the different avenues by which NPs may impact the innate immune response against bacteria. NPs (black dots) may interfere with the activation of innate cells (e.g., macrophages) by bacteria (green) in different negative and positive ways: (I) NP coating of bacteria can inhibit bacterial uptake into cells; (II) NP coating of bacteria can mask surface PAMPs and activation of cellular PRR; (III) NPs may compete with bacteria for cellular phagocytosis; (IV) NPs may have bactericidal activity resulting in release of PAMPs (small green polygons) leading to (V) PRR activation; (VI) NPs may induce epigenetic modifications (nucleosomes depicted in magenta); (VII) NP-mediated cytotoxicity causing cell membrane disruption and cell or tissue leakiness for bacterial invasion; and (VIII) NPs may enhance bacterial growth.

NPs can interact with bacteria in multiple ways, thereby interfering with the bacterial capacity to activate innate immune responses (Figure 2). It should be noted that small NPs (≤30 nm) may adhere to the bacterial surface, blocking as much as 80% of the bacterial surface area from contact with target cells and subsequent cell activation [76]. NP coating could thus serve the dual purpose of inhibiting bacterial infectivity and motility and masking the PAMPs to which TLRs or NLRs would otherwise bind [77]. It has been shown that pretreatment with Au or SiO_2_ NPs can inhibit killed *E. coli* phagocytosis by RAW 264.7 cells [78], although this may not in fact alter the course of an inflammatory response to *E. coli* infection [79]. Similarly, SPIO NPs could decrease uptake of killed *Staphylococcus pneumoniae* in bone marrow-derived mouse macrophages [56]. The general evidence that NPs inhibit bacterial phagocytosis leads to the hypothesis that they can also inhibit innate cell activation by bacteria. Indeed, data from our group show that pretreatment with Au NPs decreases the response of primary human monocytes to live Bacille Calmette-Guérin (BCG, a strain of *Mycobacterium bovis* used as a tuberculosis vaccine) in terms of cytokine production, although the NPs had no effect on LPS stimulation [59]. The mechanism remains unclear.

While the aforementioned studies describe models using safe and biomedically relevant particles, toxic NP types could enhance the detrimental impact of bacterial infections. Especially in the case of occupational exposure, disruption of tissue homeostasis by toxic NPs could open the door for pathogenicity. Particles resulting from welding fumes were observed to be particularly dangerous in this regard, with exposure driving inflammation and eventually increasing susceptibility to pneumococcal disease [80,81]. Likewise, inhalation of toxic copper oxide (CuO) NPs impaired the mouse capacity to clear a lung infection by *Klebsiella pneumoniae* [82]. While these NPs do not have a direct synergistic effect on bacterial growth, it is clear that agents that cause tissue damage and impair immune functions facilitate rapid bacterial growth and infectious spread [83,84]. The possibility that certain NP types could have a direct positive influence on bacterial growth should not be ignored [85], although data on the topic remain scant.

The bactericidal capacity of certain NPs is the most commonly reported scenario in which NPs can contribute to an effective immune reaction. Live bacteria induce immune responses of greater magnitude than their killed counterparts [9,86]. It can thus be hypothesized that the impact of NPs on immune activation caused by live bacteria could be far different than that on responses induced by PAMPs or killed bacteria. Silver NPs are perhaps the best studied particles in this regard. Ag NPs have a potent bactericidal activity mainly due to the release of toxic Ag ions that negatively impact membrane permeability and respiration [87,88]. Moreover, Ag NPs have also been found to enter the bacterial cells, where the high reactivity of silver may interfere with processes relating to sulphur (abundant on cell membranes) or phosphorus (abundant in compounds such as DNA) containing compounds [87,88]. More recently, Ag NPs have been demonstrated as effective antibiofilm agents, although it is unclear whether the particles act at the biofilm level or exclusively on bacteria [89]. Dependent upon the NP type, NPs could also contribute to biofilm formation [90]. The antimicrobial activity of other NP types, including ZnO, iron oxide, and mesoporous silica, is extensively reviewed elsewhere [91,92,93]. The use of NPs as antimicrobial agents is of great interest, in particular in the case of antibiotic-resistant infections [93]. Inhibition of bacterial proliferation and subsequent killing of pathogenic bacteria is the objective of treatment against bacterial infection, and NP-based treatments are already in use in this regard [94]. However, in scenarios such as bacterially driven sepsis, bacteriolysis following certain antibiotic treatments is known to liberate membrane-bound bacterial components such as LPS leading to excessive pathological inflammation due to uncontrolled PRR activation [95]. The same could be a pitfall for NP-caused bacteriolysis.

Direct comparison between PAMP-induced responses and responses to live bacteria is not easy, since the two responses are different. In any case, it is clear that NPs can impact both types of reaction, and that the effect of NPs on PAMP-induced innate activation is not necessarily predictive of their impact on the response to live bacteria. The possibility of using unfunctionalized NPs for modulating innate reactions to bacteria in a beneficial direction is however promising and opens the way to future applications. Table 2 summarizes some recent findings on the subject.

## 4. The Long-Term Effects of NP Exposure

In addition to the possibility that NPs can directly modify immune responses toward bacteria, several indirect and potentially long-term immune consequences of the NP–bacterial interaction should be considered. These include the possibility of improving the immune response in vaccination, the possibility of modulating resident microbiota towards improving human health, and the possibility of inducing/modulating innate memory towards increased resistance to infections.

### 4.1. NPs and Vaccines

Vaccines are among the most significant innovations in modern medicine. Successful vaccination involves two components: (i) presentation of antigen by professional antigen-presenting cells (APCs, typically dendritic cells) to induce adaptive immunity and long-term memory and (ii) innate immune activation, which drives crosstalk between innate and adaptive immune cells, facilitating and amplifying induction of adaptive immunity and memory. NPs can be applied for both functions, i.e., as carriers for improving antigen delivery, and as adjuvants that amplify immune responses and subsequent memory establishment [26,101,102,103]. 

NPs can be used to transport and deliver diverse types of antigens such as nucleic acids, proteins, peptides, and immune stimulating agents (adjuvants). Specific NP types have been reported to improve antigen processing and uptake, and prevent premature proteolytic degradation of protein antigens [104,105]. Vaccine antigen can be delivered to the target cells by either encapsulation within NPs or by antigen adsorption onto the particle surface. Encapsulation can prevent premature antigen degradation and achieve sustained release, whereas surface adsorption can both stabilize the antigen and facilitate the uptake by APCs through surface receptor-mediated mechanisms [20]. Importantly, intracellular delivery can be tuned so as to achieve presentation of the same antigen both in class I and in class II major histocompatibility complexes, in order to achieve a more complete protective immunity [106]. In addition, the particulate nature of NPs endows them with adjuvant capacity, i.e., the capacity of promoting a localized innate reaction while antigen presentation takes place. All these desirable properties can improve the vaccine delivery and efficacy compared to the other conventional delivery and adjuvant systems [26].

NP types such as inorganic NPs and polymeric NPs have been shown to be efficient antigen carriers. Immunity against *Mycobacterium tuberculosis* could be enhanced in mice using chitosan NP coated in lipid antigen, enhancing the delivery of antigen to the APCs [107]. Likewise, conjugation of N-terminal domains of flagellin onto Au NPs elicited higher titers of antigen-specific antibodies in mice compared to the carrier-free antigen [76]. Au NPs have also been used as carriers to enhance immunogenicity of antibacterial vaccines against *Yersinia pestis,* and *S. pneumoniae* [108,109,110]. Vetro et al. demonstrated that Au NPs coated with synthetic oligosaccharides corresponding to the repeating units of *S. pneumoniae* triggered better response to the oligosaccharide epitope (usually poorly immunogenic) and showed similar antibody production in vivo in the mouse as the human PCV13 pneumococcal vaccine (in which diphtheria toxoid is used as a carrier of the pneumococcal polysaccharides) [108].

Furthermore, NPs can be used either directly as adjuvants, or as adjuvant carriers concurrent with antigen. A cationic liposome-based adjuvant stabilized with a synthetic glycolipid (CAF01) could induce a strong and persistent Th1 response to tuberculosis in humans [111] and persistent protective immunity in mice [112]. In the mouse, the adjuvant properties of CAF01 was linked to protracted uptake and activation by dendritic cells [113]. PLGA particles in particular show promise for delivery of multiple molecules simultaneously. By loading PLGA nanoparticles with both TLR4 and TLR7 agonists, Kasturi et al. could demonstrate a synergistic adjuvant effect that greatly enhanced IgG antibody titers against ovalbumin compared to delivery of a single adjuvant [114]. This underlines the potential of using NPs to modulate innate immune responses for the optimal adjuvant effect, and indicates the possibility of fine tuning vaccination strategies against bacterial infection.

In summary, NP-based vaccine formulations are a promising strategy that can induce long-lasting protective memory, as NPs can function as carriers to modulate antigen delivery and improve immunogenicity, and they can also act as adjuvants that amplify the induction of protective immunity by a controlled amplification of innate/inflammatory reactions. Developing nanovaccines with optimal safety and efficacy will be of great importance, especially now, in the era of antibiotic resistance.

### 4.2. NPs and Microbiota

**Figure 3:**
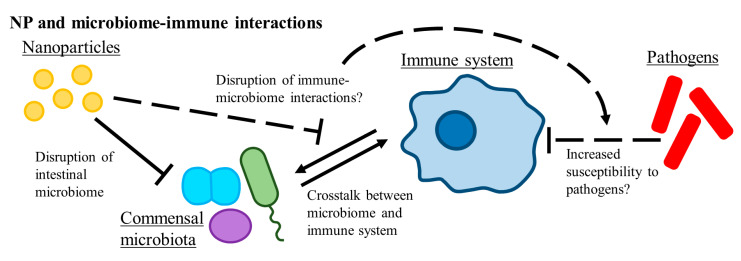
Potential disruption of host microbiota by NPs could lead to altered host immune responses and increased pathogen susceptibility. Solid arrows indicate interactions that are well described in literature. Dashed arrows indicate understudied interactions and possible future focal points for NP research.

Perhaps the most likely avenue for NP interactions with bacteria in a human context occurs within the several locations inhabited by microfloral populations. Figure 3 depicts some potential implications of such NP–bacterial interactions on human innate immunity. The gastro-intestinal tract is colonized by a large and highly interactive community of microbes that play a pivotal role in host health by providing essential nutrients and aiding in digestion. It is now increasingly recognized that these commensal microbiota also contribute to the host immune defense, for example, by providing resistance against invading pathogens and by training and stimulation of the host immune system, as reviewed by [73,115,116]. Owing to these critical functions provided by intestinal microbiota, their disruption (dysbiosis) by, for example, a dietary change or a chemical exposure may adversely affect the health of the host [117,118,119] and in humans is associated to numerous diseases including obesity, inflammatory bowel disease, and diabetes [120]. Given the central role that microbiota play in host immunity and host health, there is a need to incorporate commensal microbiota in the health risk assessment of NP applications, both from a biomedical standpoint, and in the context of occupational, consumptive, or inadvertent NP exposure [121].

An increasing body of literature now indicates that NPs have the potential to disrupt both the gastro-intestinal, but also the respiratory microbiota of animals, as reviewed by [74,118,122,123,124,125]. Some studies have indicated that in rodents Ag NPs can negatively affect *Lactobacillus* and other core intestinal *Firmicutes* [126,127]. These results are, however, contrasted by other findings that show no or a positive impact of Ag NPs on the relative abundance of these bacterial taxa in the intestinal microbiota of rodents [128,129,130]. Exposure to micro- and nanoplastics has also been proposed as a potential cause of gut dysbiosis [131,132]. Further investigation will help reveal the extent by which NPs can disrupt the commensal microbiota and the consequent impact of NPs on gastro-intestinal conditions.

Further, it remains unclear which implications dysbiosis induced by NPs could have on host health [122,125]. Some studies have shown that microbiota modulations under NP exposure coincide with changes in the expression of host immune markers [126,127,133], which may reflect the inherent link between microbiota and host immunity. In contrast, other studies have found no relation between microbiota changes and host immune status under NP exposure [134]. A modulation of the expression of immune markers by NPs does not necessarily indicate that the host is being immunocompromised. However, a chronic stimulation of intestinal epithelial cells consequent to synergistic NP-microbial effects and leading to a chronic inflammatory milieu in the gut may have critical consequences on barrier integrity and long-term tissue homeostasis. To study whether NPs actually have an adverse effect on the host immune status through dysbiosis, we need animal models in which immunity to infections or other challenges is assessed [134,135]. To date, the possible impact of NPs on the interactions between host immunity, microbiota, and host health status remains largely unexplored, but given the widespread exposure to NPs this clearly requires further investigation.

### 4.3. NPs, Bacteria, and Innate Immune Memory

It is now evident that innate immune cells can develop memory of past challenges, which makes them able to react to subsequent stimulations in a more efficient and more protective fashion. Some stimuli, most notably LPS, drive a tolerance-type memory, in which production of inflammatory effectors is less potent in response to a second challenge, in order to achieve protection without risking the significant tissue damage that a strong reaction to LPS would cause [136,137]. Conversely, in response to other agents (e.g., fungal β-glucan, oxidized low-density lipoproteins, and BCG) a different type of memory develops, which leads to an increase in cellular reactivity upon a second challenge, again aiming at improving health protection [138,139,140]. This elevated response has been termed “trained immunity” or “potentiation” [141,142]. Thus, innate immune memory is a protective mechanism, with tolerance shielding the host from excessive inflammation and consequent tissue damage [140], and potentiation enhancing the host defense against unrelated pathogens [143]. Moreover, it appears that innate immune memory may be conferred either locally, for instance within resident macrophages of the lung [144], or systemically via monocyte progenitors in the bone marrow [145,146]. In both scenarios, innate immune memory is a long-lasting phenomenon (life-long in invertebrates, at least a few months in humans), despite the shorter lifespan of memory monocytes and macrophages. Most importantly, at variance with immunological memory in adaptive immunity, innate memory in mammals (including human beings) is largely non-specific, meaning that priming (first stimulation) with an agent such as BCG may induce a more powerful secondary response to an unrelated stimulus. Thus, innate memory is apparently one of the mechanisms at the basis of the non-specific protection induced by vaccination/priming that enhances resistance to vaccine-unrelated diseases [147,148,149]. 

Since engineered NPs are foreign particles that the innate immune system may recognize as potentially dangerous, it is well possible that they could act as innate memory inducers and/or interfere with memory induction by bacteria and other stimuli. Two scenarios should be considered: (i) that unintentional exposure to NPs or microorganisms together with NPs may erroneously prime the innate immune system for inadequate response to future challenges (e.g., by provoking excessive and destructive inflammation to a subsequent infection) and (ii) that NPs could be used to deliberately induce/modulate innate memory in order to prime the innate immune system towards a more efficient response to future infections, in a sort of non-specific innate vaccination.

In 2017, the first evidence that NPs can induce innate memory was published, which showed a memory effect induced by Au NPs on human primary monocytes and that also hypothesized that the NP-dependent epigenetic reprogramming capacity could be at the basis of innate memory induction [150]. In 2020 several independent reports showed that different NP types could induce innate memory. In the marine bivalve *Mytilus galloprovincialis*, previous exposure to nanoplastics modulated the hemocyte subpopulations and immune-related genes, resulting in an increased bactericidal capacity upon subsequent exposure to nanopolystyrene [133]. This is the first indication that NPs, similar to bacteria and PAMPs, could induce an innate memory resulting in increased resistance to subsequent infections. Another study demonstrated that pristine graphene (unable to induce cell activation capacity per se) could prime mouse bone marrow-derived macrophages to react to a subsequent challenge with different PAMPs (LPS, CpG, and R848) with an increased production of the inflammatory cytokines IL-6 and TNFα, in parallel to a decreased production of the anti-inflammatory cytokine IL-10, indicating graphene-induced memory reprogrammed macrophages in the direction of immune potentiation, despite the apparent inert nature of the nanomaterial [151]. In addition to the capacity of some NPs to induce innate memory, an interesting possibility is that the presence of NPs may modulate the priming/memory-inducing capacity of other agents. Preliminary results indeed show that the memory response of human primary monocytes primed with live BCG and challenged with LPS, in terms of production of inflammatory TNFα and IL-6 and anti-inflammatory IL-1Ra and IL-10 cytokines was significantly reduced if Au NPs were present with BCG during priming [59]. These findings open the possibility of novel approaches of immunomodulation, in which NPs can be used for improving vaccine efficacy and general resistance to infections, and also for modulating and rebalancing the altered immune responses in a range of immune-related diseases, such as chronic inflammatory, degenerative, and autoimmune diseases [152].

## 5. Conclusions and Future Perspectives

Human exposure to NPs has become increasingly frequent in modern society, both from an occupational and consumer standpoint, and in biomedical applications. Here we addressed the impact that NPs may have on the innate immune response, in particular within the context of the protective response to live bacteria. NPs can interfere with bacterial life, motility, growth, and biofilm formation, and can decrease phagocytosis of bacteria by human immune cells. Since the immune reaction to live bacteria is much more complex than that to PAMPs, generally used in vitro as a bacterial proxy, reliable assays for assessing the capacity of NPs to affect the innate immune response should make use of live bacteria. It also becomes apparent that the NP impact on innate responses to bacterial infection is not limited to the immediate reaction. In vaccination strategies, NPs can facilitate the development of long-lasting specific immunity against pathogenic bacteria at multiple levels: by improving antigen delivery to antigen-presenting cells, by acting as adjuvants that amplify adaptive responses through a controlled inflammatory reaction at the site of antigen presentation, by activating antigen-presenting cells for more efficient antigen presentation, and through modulation of innate memory, which could prime innate immune cells towards a more efficient response to the vaccine. Moreover, NPs may alter the composition of the host microbial community, potentially altering synergism between the host and microflora during immune responses. In each case, in order to understand the full potential impact of NP exposure on human health and innate immunity we need to examine their interactions with live bacteria in suitable in vitro and in vivo models. Overall, based on the most recent data, the possibility of using NPs for modulating innate immune responses towards increased resistance to infections is a promising and realistically feasible possibility.

## Figures and Tables

**Table 1:** Overview on studies investigating the effect of NPs on innate immune cell activation.

NP Type (Size, Dose, Shape)	Stimulus (Receptor)	Effect on Stimulus-Induced Innate Response			Cell Type	Notes	Discusses NP Dose Selection	Ref
		↑	↓	=				
Au 5–35 nm 10–70 µg/mL not available (n/a)	IL-1β (IL-1R1)		×		Human THP-1 monocyte-like leukemia cells	Induction of cytokine production	No, some experiments addressed surface area	[55]
	R848 (TLR7/8)			×				
25 nm 10 µg/mL spherical	LPS (TLR4)			×	Human primary monocytes	Induction of cytokine production	Yes, endotoxin-free concentrations used	[59]
Au (10 nm), Ag (14 nm) 1.3–12 µg/mL spherical	LPS + TNFα (TLR4+TNFR)			×	Human primary monocytes	Kinetic evaluation of cytokine expression and production	Yes, concentrations chosen based on cytotoxicity and endotoxin assays	[6]
Pt 2.4 nm 10–1000 µM n/a	LPS (TLR4)		×		Mouse RAW 264.7 macrophage-like leukemia cells	Induction of cytokine and ROS production	No, use of different concentrations not discussed	[53]
Iron oxide 58.7 nm 1–50 µg/mL n/a	LPS 100 ng/mL (TLR4)		×	×	Primary murine microglial cells	Particle accumulation in lysosomes, decreased IL-1β but not TNFα production	Utilized a dose response	[60]
n/a 0–30 mg/mL n/a	Apoptotic cancer cells (Scavenger R)	×			Mouse RAW 264.7 macrophage-like leukemia cells in coculture with other cancer cells (in vitro); tumor-associated macrophages (in vivo)	In vitro: M1 cell polarization In vivo: M1 polarization at day 7; M2 polarization at day 21	Yes, doses chosen were related to human administered doses of ferumoxytol	[61]
10, 30 nm 1–100 µg/mL spherical	LPS (TLR4)		×		Human primary monocytes	Induction of TNFα, IL-6 and IL-1β production	Utilized a dose response	[54]
11 nm 2 µg/mL spherical	LPS + TNFα (TLR4+TNFR)			×	Human primary monocytes	Kinetic analysis of expression and production of IL-1β and IL-1Ra	Yes, selected the highest endotoxin-free concentration	[62]
Lipid-modified glycol-split heparin 110, 160 nm 0.5 mg/mL spherical	LPS (TLR4), PAM3CSK4 (TLR1/2), Poly(I:C) (TLR3)		×	×	Mouse peritoneal macrophages	Signal transduction and cytokine production inhibited only in LPS-activated cells	Utilized a dose response	[63]
PCL-PEG 70–130 nm 1–100 µg/mL n/a	LPS (TLR4)			×	Human primary monocytes	Induction of TNFα and IL-1β production	NP concentration equalized for surface area, no explanation for dose selection	[58]
Pristine graphene 100–1000 µm 20–100 µg/mL crystalline	LPS (TLR4) in vitro, heat killed *E. coli* ex vivo	×		×	Mouse ex vivo peritoneal macrophages and bone marrow-derived dendritic cells	NLRP3 inflammasome activation and IL-1β production (increase), IL-6 and IL-12p70 production (unchanged)	Yes, sub-toxic dose selected	[64]
Carbon black 14, 56 nm 4 mg/kg n/a	LPS (TLR4)	×			In vivo mouse lung	Lung inflammation (histology) and cytokines production (IL-6, TNFα)	Referenced previous work	[65]
CeO_2_ 3–5 nm 1 nM–10 µM crystalline	LPS + IFNγ		×		Mouse J774.A1 macrophage-like histiocytic lymphoma cells	ROS production, iNOS protein production	Yes, dose responses were used	[66]

IL-1R1: Interleukin 1 receptor 1; TNFR: TNF receptor; Pt: platinum; PCL: poly(ε-caprolactone); PEG: polyethylene glycol; ROS: reactive oxygen species; INOS: inducible nitric oxide synthase.

**Table 2:** Overview on studies on the impact of NPs on innate immune stimulation by live bacteria.

NP Type (Size, Dose, Shape)	Stimulus	Effect on Bacteria- Induced Response			Cell Type	Notes	Discusses NP Dose Selection	Ref
		↑	↓	=				
Au 13 nm 0.17–17 mg/kg spherical	*E. coli*			×	In vivo mouse	Intravenous delivery daily during the course of the infection	No	[79]
25 nm 10 µg/mL spherical	BCG		×	×	Human primary monocytes	Endpoint: production of TNFα, IL-6 and IL-10 (inhibited), IL-1Ra (unchanged)	Yes, endotoxin-free concentrations used	[59]
Au 10, 300 nm 5, 10 mg/mL spherical SiO_2_ 10, 300 nm 5, 10 mg/mL spherical	*E. coli*		×		Mouse RAW 264.7 macrophage-like leukemia cells	Pretreatment with Au and SiO_2_, cells displayed reduced phagocytosis of FITC labeled *E. coli* (no inflammation data for bacterial exposure)	Yes, dosimetry data based on relevant dose metrics (by area, by number, by volume) specifically designed for a functional study, checked for artificial overdosing; no relation to human exposure	[78]
SiO_2_ 30, 140 nm 25% bacterial coverage spherical	*E. coli,* *H. pylori*		×		Human AGS stomach adenocarcinoma cells	Endpoint: IL-8 production. Bacteria precoated with NPs	Yes, based upon predicted bacterial surface coverage	[76]
50.9 nm 5 mg/kg n/a	*P. aeruginosa*	×		×	In vivo mouse NP lung pretreatment followed by challenge	Endpoints: cytokine production (IL-6, KC, IL-1β, IL-12, TNFα) and bacterial phagocytosis (unchanged), mortality (increased)	No; potential for overdosing with invasive and unphysiological nasal instillation; yes, in relation to a mechanistic study	[83]
Ag 80 nm 10 µg/mL n/a	*S. aureus, E. coli*	×		×	Human monocyte-derived macrophages, osteoclasts	Endpoints: intracellular bactericidal activity, ROS generation	Yes, with dose determination for a functional study and without relation to human exposure	[96]
Ag-PVP 10, 20, 80 nm 0.78–200 µg/mL n/a	*C. trachomatis*		×		Mouse J774 macrophage-like histiocytic lymphoma cells	Endpoint: IL-6, TNFα and other cytokines and chemokines)	Yes, dose response to determine maximal effective concentration	[97]
Iron oxide 100 nm 3 mg/mL n/a	*S. aureus*	×			Mouse RAW 264.7 nacrophage-like leukemia cells, topical application of NP or bacteria on the mouse	in vitro: IL-1β, TNFα, IL-12 production, in vivo: CFU following infection (decreased), cytokine expression (IL-1β increased)	Utilized a dose response	[98]
13 nm 6.25–50 µg/mL spherical)	*S. pneumoniae*	×			Mouse bone marrow-derived macrophages	NP pretreatment resulted in decreased bacterial phagocytosis	Yes, allegedly mimicking occupational exposure doses (not explained how); particle kinetics in culture discussed and macrophage overload tested	[56]
CuO 12 nm 3–100 µg/mouse core/shell	*K. pneumoniae*	×			Murine model of lung infection	NP toxicity, inflammation (IL-6, TNFα, KC, others), and reduced ability to clear bacteria	Yes, for sub-acute inhalation dose range mimics human occupational exposure for intratracheal instillation 3 concentrations with dose bridging to human inhalation exposure	[82]
Diesel exhaust particles n/a 80 µg/mouse n/a	*S. pneumoniae*	×			Murine model of lung infection	Mouse more susceptible to pathogenic infection, increased lung homogenate cytokines (IL-6, TNFα, IL-1β, KC, others)	No rational given	[99]
Welding fumes 100–1000 nm 600 µg/mouse n/a	*S. pneumoniae*	×			Murine model of lung infection	Increased bacterial CFU in exposed lungs	No rational beside use of a high dose	[100]

*H. pylori*: *Helicobacter pylori*; KC: mouse CXCL1; PVP: polyvinylpyrrolidone; *P. aeruginosa*: *Pseudomonas aeruginosa*; *C. trachomatis*: *Chlamydia trachomatis*; CFU: colony forming units; *S. pneumoniae*: *Staphylococcus pneumoniae*; *K. pneumoniae*: *Klebsiella pneumoniae*.
